# Participation and quality of life in children with Duchenne muscular dystrophy using the International Classification of Functioning, Disability, and Health

**DOI:** 10.1186/1477-7525-10-43

**Published:** 2012-05-22

**Authors:** Roxanna M Bendixen, Claudia Senesac, Donovan J Lott, Krista Vandenborne

**Affiliations:** 1 Research Assistant Professor, Department of Occupational Therapy, University of Florida, Gainesville, FL, 32611, USA; 2 Clinical Assistant Professor, Department of Physical Therapy, University of Florida, Gainesville, FL, 32611, USA; 3 Research Assistant Professor, Department of Physical Therapy, University of Florida, Gainesville, FL, 32611, USA; 4 Chair, Department of Physical Therapy, College of Public Health & Health Professions, University of Florida, Gainesville, FL, 32611, USA

**Keywords:** Duchenne muscular dystrophy, ICF, Participation, Quality of life

## Abstract

**Background:**

Duchenne muscular dystrophy (DMD) is characterized by muscle damage and progressive loss of muscle function in male children. DMD is one of the most devastating genetically linked neuromuscular diseases for which there is currently no cure. Most clinical studies for DMD utilize a standard protocol for measurement exploring pathophysiology, muscle strength and timed tasks. However, we propose that examining broader components of health as emphasized by the International Classification of Functioning, Disability and Health-Children and Youth Version (ICF-CY) may be of great value to children and their families, and important outcomes for future clinical trials.

**Methods:**

Fifty boys with DMD and 25 unaffected age-matched boys completed two self-report measures: the Children’s Assessment of Participation and Enjoyment and the Pediatric Quality of Life Inventory^TM^ 4.0. We investigated differences between the two groups with regard to participation in life activities and perceived quality of life (QoL). Additionally, we compared participation in activities and QoL in both cohorts of younger and older boys.

**Results:**

Participation in physical activities was significantly lower in boys with DMD than unaffected boys. Perceived QoL was markedly diminished in children with DMD relative to unaffected controls, except in the emotional domain. The amount of time boys engage in an activity, as well as participation in social activities, declined for our older boys with DMD but no changes were observed for our older unaffected boys. For both groups, QoL remained constant over time.

**Conclusions:**

The ICF-CY provides a conceptual framework and specific terminology that facilitates investigation of the consequences of impairment in children and youth. Our study is one of the first to explore participation in a cohort of boys with DMD. It was not surprising that activities of choice for boys with DMD were less physical in nature than unaffected boys their age, but the consequences of less social engagement as the boys with DMD age is of great concern. Results from our study underscore the need to further evaluate activities that children elect to participate in, with special emphasis on facilitators and barriers to participation and how participation changes throughout the course of a disease.

## Background

Duchenne muscular dystrophy (DMD), the most common form of muscular dystrophy, is a genetically-linked disease of male children which affects one in 3,300 boys [1]. The muscle damage and weakness which occurs with this disease leads to a progressive decline in the boy’s ability to perform everyday tasks, such as climbing stairs and walking short distances [2-4]. Disruption to the lives of children with DMD and their families begins as early as age three when muscle weakness interferes with daily activities. As the weakness progresses, the boys often become confined to wheelchairs early in the second decade of life [5]. End stage cardiac and respiratory complications lead to death which frequently occurs around the age of 20 [6-8]. Overall, DMD leads to a loss of functional independence and deterioration in the quality of life for both the children and their families [9-13].

It is imperative to develop techniques which can be used to follow the progression of this disease as well as the efficacy of any therapeutic interventions. Traditional measures used to follow DMD are typically clinical measures of skeletal muscle structure and function [14-17]. These outcome measures include muscle strength, manual muscle testing, range of motion, forced vital capacity, anthropometric tests, timed functional tests and the 6 min walk. In addition, there are also invasive techniques, such as muscle biopsy, and noninvasive techniques, such as magnetic resonance imaging, which have been used to follow the structural and compositional changes that occur with the progression of DMD [18,19]. Although these measures represent the current models on which clinical progression of the disease is based [20,21], such measurements may not fully capture the impact of the disease on real life activities. The medical community has long assumed that disability directly correlates with the measurements made in a clinical setting. However, it has been shown that measures of pathophysiology and motor function assessed in the clinic do not necessarily relate to or predict how patients deal with life situations [22-24]. Clinical measures are often disconnected from the context and synergy of everyday life. Therefore, perhaps other nontraditional outcome measures would also be valuable in following the progression of DMD.

**Figure 1:**
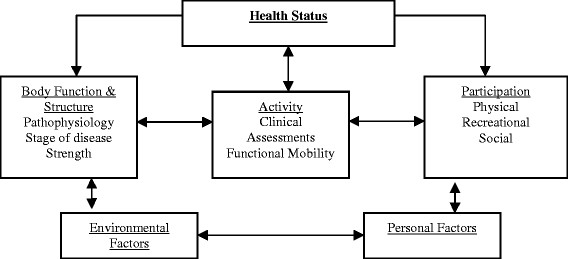
International Classification of Functioning, Disability and Health-Children and Youth Version (ICF-CY).

One outcome measure which could be used to evaluate the ability of boys with DMD to manage real life situations is to determine their degree of *participation* in various activities. The International Classification of Functioning, Disability, and Health-Children and Youth Version (ICF-CY) adopted by the World Health Organization (WHO) [25] (Figure 1) identifies participation as an important outcome of health. Participation is defined as direct engagement in a life situation including activities of personal care, mobility, social relationships, education, recreation and leisure, spirituality, and community life [25,26]. It has been reported that children with physical disabilities engage in less varied activities than healthy children [27-30]. To the best of our knowledge, participation levels among children with DMD and changes in the patterns of participation with age and disease progression have not been investigated. Therefore, there is a need to quantify participation in boys with DMD and the effect participation may have on their quality of life throughout the course of the disease.

Quality of life (QoL) measures have often been used in the healthcare field to compare diseases, determine the effects of an illness [31], and measure the outcomes of treatments and clinical trials [32,33]. It is possible that QoL measures can shed new insight into patient outcomes that traditional physical measures may not be able to assess [11,33-35]. It has been reported that children with chronic illnesses typically score below healthy children in QoL assessments, although patients with progressive diseases such as DMD have been excluded from most comparative studies [36,37]. Surprisingly, there is little information on the QoL of children with DMD despite parents reporting quality of life issues as an important challenge for their children [38,39]. The progressive decline in strength and loss of the ability to participate in activities with their peers can lead to social exclusion and isolation for these boys. These factors may have a significant impact on how boys with DMD perceive their quality of life.

The objectives of this investigation were 1) to compare boys with DMD with an unaffected age-matched population of boys with regard to participation in real life activities and perceived quality of life, 2) to determine the differences in participation in activities and perceived quality of life in younger and older boys with DMD and unaffected boys to explore the effects of disease progression, and 3) to determine if there is a relationship between participation in activities and perceived quality of life in younger and older boys with DMD and unaffected boys. This was accomplished by using two self-report outcome measures, the Children’s Assessment of Participation and Enjoyment (CAPE) and the Pediatric Quality of Life Inventory^TM^ 4.0 (PedsQL).

## Methods

In our study we collected data from a cohort of boys participating in a larger natural history study exploring biomarkers of disease progression in Duchenne muscular dystrophy. Our study investigated self-report of participation levels and QoL in boys with DMD compared to unaffected age-matched boys. Fifty boys with DMD (validated through medical report using molecular genetic testing and/or immunohistochemical staining from muscle biopsy) and their parent(s) volunteered to enroll in this study. Twenty-five unaffected age-matched boys were recruited from the general population for comparisons in participation and QoL. Unaffected boys were relatively sedentary, in that they did not participate in sport specific training more than 2 times per week. All boys and their parents participated in person at our site. The boys ranged in age from 5 to 15 years. Three boys with DMD who were nonambulatory were included in this study; all others were ambulatory at the time of testing. The study was approved by the University of Florida Institutional Review Board (IRB 129-2008). Written, informed consent was obtained from the parent(s), and written assent was obtained from each participant. Following informed consent, the boys completed two paper/pencil self-report assessments which focused on participation in physical, recreational and social activities and perceived quality of life. Assessments of participation were completed with the assistance of their parent(s).

### Instruments

Participation was measured by the **Children’s Assessment of Participation and Enjoyment (CAPE)**[40]. The CAPE is designed to examine how children and youth participate in everyday activities. Scores on the CAPE are divided into five dimensions: 1) Diversity, the number of activities performed overall; 2) Intensity, the frequency of performing the activity; 3) With Whom the activity is performed; 4) Where the activity is performed; and 5) how much Enjoyment the child gets from the activity. Additionally, activities are divided into the following domains: physical, recreational, social, skill-based, and self-improvement. Three activity domains of the CAPE were used in our calculations (physical, recreational and social activities). Physical activities included such items as playing team sports, bicycling, and actively using playground equipment. Recreational activities included reading, playing cards or playing video/computer games. Social activities included visiting friends, going to the movies, and cooking. Reliability and validity of the CAPE were established using data from a longitudinal study involving 427 children with physical disabilities [27]. Although validation studies with the CAPE did not include children with progressive diseases, such as DMD, other diagnoses which result in muscle weakness and motor difficulties were included (i.e., cerebral palsy, amputation, muscular disorders, neuropathy, orthopaedic conditions, vascular brain disorders). In addition, the CAPE was designed to be a direct measure of participation as reflected by the WHO’s ICF model [27]. The CAPE documents what a child does in their everyday life, not the child’s competence or the degree of support required to participate in the activity [28]. Therefore it was felt the CAPE measure would adequately capture the boys with DMD involvement in life situations.

**Quality of Life (QoL)** was assessed through the **Pediatric Quality of Life Inventory^TM^ 4.0 (PedsQL),** which is designed to measure the core dimensions of health as delineated by the WHO (physical, emotional, social), as well as school functioning, and has been determined to be a thorough and reliable measure of QoL in children [36,37,41]. Generic quality of life instruments have frequently been used as patient reported outcome measures, and their use encouraged to explore the clinical meaningfulness of a change in more traditional clinical measures of body systems and overall function [26,33]. Generic QoL measurements provide valuable information for comparing populations, but may not be sensitive enough to detect change necessary for a treatment based clinical trial [42]. The PedsQL^TM^ 4.0 has previously been used to evaluate patient-perceived well being in boys with DMD and unaffected controls [43] and to investigate agreement between boys with DMD and their parents when reporting quality of life [13,44]. The data for the Generic Core Scales were derived from the PedsQL^TM^ 4.0 field test [37]. Generic Core Scales included children who are healthy, acutely ill, and chronically ill.

### Statistical analysis

All statistical analyses were performed using statistical software PASW version 18.0 for Windows (PASW Inc., Chicago, IL). The Mann–Whitney *U*-test was used for group comparisons and the Spearman’s rank correlation coefficient test was used for non-parametric analysis of correlations. A p-value of less than or equal to 0.05 was considered statistically significant. In our aim of exploring the differences in participation in life activities and perceived QoL between younger and older unaffected boys and boys with DMD, a p-value of less than or equal to 0.01 was considered statistically significant due to the multiple number of analyses performed.

## Results

### Characteristics of boys with DMD and unaffected age-matched boys

A comparison of the characteristics of the boys with Duchenne muscular dystrophy (DMD) and the unaffected age-matched boys used in this study is shown in Table 1. The ages and weights of the boys in both groups were comparable. However, boys with DMD were shorter in height and had a higher body mass index (BMI) than unaffected boys. One possible reason for the decrease in height and higher BMI may be the fact that all boys in the DMD group were taking steroids. It is known that administration of steroids may result in weight gain and short stature [45]. A comparison of the ability to participate in life activities and their perceived QoL was made between these two groups, and the results are presented below.

**Table 1:** Characteristics of DMD and unaffected age-matched subjects

	**DMD (n = 50)**	**Unaffected (n = 25)**
Age	8.9 (2.4)	8.4 (2.1)
Weight (kg)	32.3 (10.5)	31.1 (10.2)
Height (m)	122.6 (12.5)	142.5 (11.3)**
BMI (kg/m^2^)	19.5 (3.4)	16.7 (3.4)*

* p ≤ .05; **p ≤ .01.

### Participation and QoL in boys with DMD Vs unaffected boys

**Figure 2:**
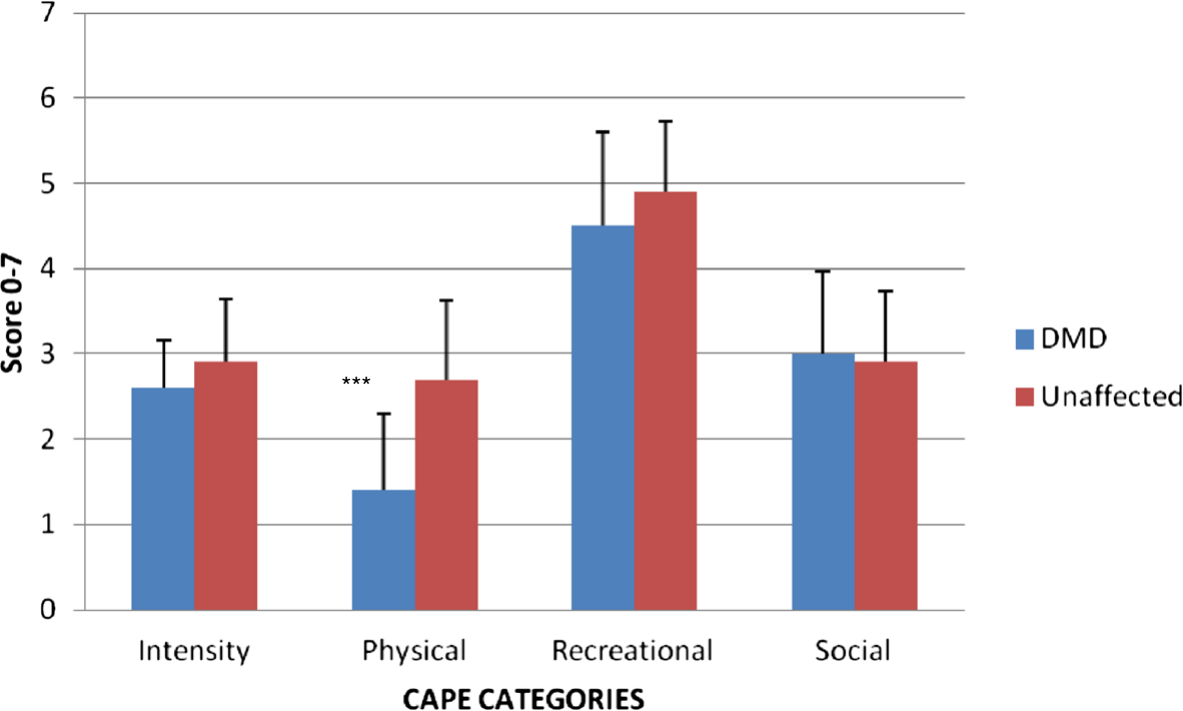
**Baseline CAPE Responses by DMD (n = 50) and Unaffected Age-Matched Subjects (n = 25).** CAPE, Children’s Assessment of Participation and Enjoyment collection instrument. DMD, Duchenne muscular dystrophy; Range of scores (Intensity 0–7; Physical Activities 0–7; Recreational Activities 0–7; Social Activities 0–7). Data are presented as means *±* standard deviations. ***p <0. 001.

Participation in life activities for each group was assessed using the Children’s Assessment of Participation and Enjoyment (CAPE), a self-report outcome measure. The results were divided into four categories (Figure 2). Intensity is a measure of the frequency of participation in chosen activities varying from no participation (score 0) to once in the last 4 months (score 1) to daily (score 7). The total number of activities in which an individual participates in (Diversity) and intensity scores are used to compute scores for the three different types of activities: physical, recreational, and social. The range of activity scores is from 0 (lowest participation) to 7 (highest participation). The results showed lower participation levels in physical activities in DMD (p < .001), but no significant differences between boys with DMD and unaffected boys in diversity or any of the other categories (intensity, recreational or social activities).

**Figure 3:**
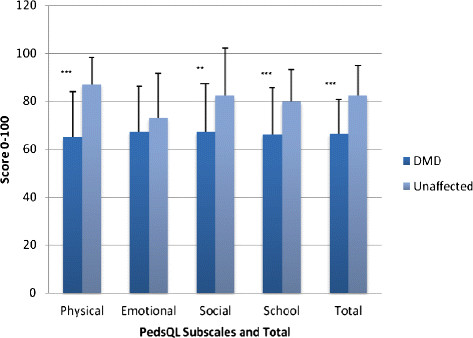
**Baseline PedsQL Responses by DMD (n = 50) and Unaffected Age-Matched Subjects (n = 25) .** DMD, Duchenne muscular dystrophy; PedsQL^TM^, Pediatric Quality of Life collection instrument; Range of scores 0–100. Data are presented as means ± standard deviations. *p <0.05; **p < 0.01; ***p < 0.001.

The perceived QoL for each group was assessed using the Pediatric Quality of Life Inventory^TM^ 4.0 ™ (PedsQL), also a self-report outcome measure. These responses were divided into four subscales: physical, emotional, social, and school-related. In addition, a total QoL composite score was also included (Figure 3). The range of scores is from zero (lowest QoL) to 100 (highest QoL). There were lower scores for boys with DMD than the unaffected group in physical, social, school-related, and total composite quality of life responses. There were no significant differences between groups in the responses related to emotional factors. These results demonstrate that, in general, the perceived QoL is diminished in boys with DMD.

### Participation and QoL in younger and older boys

To assess the effects of disease progression, the boys with DMD and unaffected boys were divided into two age groups, i.e., less than 10 years and 10 years or older. The age of 10 years was selected because of the disease progression and the muscle and functional changes that reportedly occur for boys with DMD around this age, including the ability to rise from the floor, walk up stairs or ambulate [5,17,19]. All demographics for these two age groups (age, height, weight, BMI) were statistically significantly different at p < .001. For boys with DMD, there were significant differences between the two age groups in CAPE scores of direct participation in social activities (p ≤ 0.01) and a trend toward significance in overall frequency of participation in all activities (p ≤ 0.05), but no differences in diversity of activities or participation in physical or recreational activities. No differences were observed between these two DMD age groups in self-report of QoL in any of the subscale scores or the total composite score of the PedsQL^TM^ (Table 2). These results demonstrate that overall, older boys with DMD participate in a variety of activities, but less often than their younger peers. More importantly, engagement in social activities declined significantly for our older boys with DMD. Interestingly, perceived quality of life did not vary between the two DMD age groups. For our unaffected group, no significant differences were found between the two age groups in any PedsQL ^TM^ subscale scores or total composite score (Table 2).

**Table 2:** Baseline CAPE and PedsQL^TM^ responses by DMD and unaffected based on age groups: differences within and between groups

Instruments	**DMD (n = 50)**		**Unaffected (n = 25)**		**P value Between Groups** **<10 years**	**P value** **Between Groups** ** ≥ 10 years**
	**<10 years (n = 27)**	**≥ 10 years (n = 23)**	**<10 years (n = 13)**	**≥ 10 years (n = 12)**		
CAPE Diversity	30.1 (5.2)	27.6 (5.4)	33.3 (7.1)	30.1 (4.2)	0.05	0.19
CAPE Intensity	2.7 (0.6)	2.4 (0.5)*	3.2 (0.8)	2.6 (0.5)	0.18	0.68
CAPE Physical	1.4 (0.7)	1.3 (1.1)	2.9 (0.9)	2.5 (0.9)	0.00	0.01
CAPE Recreational	4.9 (1.0)	4.6 (1.1)	5.2 (0.7)	4.8 (0.7)	0.38	0.42
CAPE Social	3.4 (0.9)	2.4 (0.8)**	3.1 (0.7)	3.0 (0.9)	0.85	0.02
PedsQL Physical	68.1 (16.7)	61.6 (21.2)	87.8 (11.5)	85.3 (12.5)	0.00	0.00
PedsQL Emotional	63.2 (17.6)	71.8 (19.8)	73.1 (18.8)	73.6 (19.1)	0.13	0.83
PedsQL Social	68.5 (22.9)	65.9 (23.5)	79.9 (21.3)	86.4 (17.9)	0.14	0.03
PedsQL School	64.5 (20.7)	67.7 (18.0)	78.9 (13.9)	81.4 (13.7)	0.18	0.05
PedsQL Total Composite	66.2 (11.4)	66.7 (16.9)	82.7 (13.1)	81.6 (12.7)	0.00	0.02

*DMD,* Duchenne muscular dystrophy; *CAPE*, Children’s Assessment of Participation and Enjoyment collection instrument; *CAPE,* Range of scores (Diversity 0–55; Intensity 0–7; Physical Activities 0–7; Recreational Activities 0–7; Social Activities 0–7); *PedsQL*, Pediatric Quality of Life collection instrument*; PedsQL*, Range of scores 0–100. Data are presented as means ± standard deviations. *p < .05; **p < .01.

Differences between the younger boys with DMD and the younger unaffected boys were observed in the CAPE area of physical participation, as well as the PedsQL^TM^ physical subscale and total composite score (all significant at p ≤ 0.000). For our older cohort (≥10 years), boys with DMD scored significantly lower in the physical and social domains of the CAPE, as well as the PedsQL^TM^ physical and social subscales and the total composite score (Table 2).

### Relationship between participation and QoL

As the results above demonstrate, participation in activities that were social in nature declined for the older cohort of boys with DMD; self-report of QoL did not differ between the two DMD or unaffected age groups. Correlational analyses showed no significant relationship between overall participation and perceived QoL in either age group of boys with DMD or unaffected boys.

## Discussion

In this study we compared participation in life activities and perceived QoL between boys with DMD and an age-matched population of unaffected boys. The results showed that participation in physical activities was significantly lower in boys with DMD than unaffected controls, although other participation measures were much the same as healthy boys. Furthermore, perceived QoL was markedly diminished in children with DMD relative to unaffected controls. Another objective of this study was to compare younger boys to older boys in both the DMD and unaffected cohorts to explore both within group and between group differences in participation and QoL. For within group differences, we found that the amount of time boys with DMD engage in an activity (frequency), as well as participation in social activities, declined for our older cohort but there was no decline in participation observed for our older unaffected boys. No differences in perceived QoL were observed between the younger and older boys with DMD or the unaffected boys. Significant between age group differences were observed among the boys with DMD and the unaffected controls. Comparisons between our younger boys with DMD and our younger unaffected boys showed lower scores in diversity of activities they participate in, as well as participation in physical activities. Quality of life scores were lower in the physical subscale as well as the overall total composite score. For our older boys with DMD, lower scores were reported in both physical and social participation, as well as quality of life subscales of physical, social and total composite scores compared to our older unaffected boys. No differences were observed in the PedsQL^TM^ subscales of emotional or school related QoL for our older boys. Finally, no correlations were observed between reports of participation and QoL in these two groups.

Our study is one of the first to explore self-report of participation in activities in a population of boys with DMD. The results demonstrated that, overall, the boys with DMD chose diverse activities in which to participate and that they were involved in their chosen activities as frequently as healthy boys. However, the activities of choice for boys with DMD were less physical in nature than unaffected boys their age. It certainly comes as no surprise that children with muscular weakness are less likely to participate in physical activities, especially as they age [46,47]. Interestingly, physical activity for healthy boys has been reported to decline during later childhood or adolescence stages [48,49], although we did not observe a decline in our unaffected group of boys. In contrast, this age-related decline in physical activities for unaffected boys does not appear to be due to the bodily demands of the activity [49,50]. Moreover, physical activity participation is typically high for younger unaffected boys [50], but was significantly lower in our younger boys with DMD.

In boys with DMD, participation in social activities, such as visiting friends, going to parties or going to the movies was significantly lower after the age of 10 years. This is often the age when boys with DMD experience a decrease in motor abilities, such as ambulation [5,46] and may, therefore, have less opportunity for community-based social engagement. It’s important to note the connection between physical abilities and social engagement. Poor motor skills have been associated with peer relationship difficulties, lower peer regard and greater likelihood of suffering from withdrawal [51]. Actively engaging in physical activities, such as team sports or playing with others on the playground, helps children form peer relationships and experience a sense of self-worth and belonging to a group [30,40,51,52]. Unfortunately, little is known about the social engagement of children with DMD. Although parents more often report prolonging ambulation as the most important factor in dealing with DMD [39,53], the resulting emotional challenges of DMD and the increasing social needs of boys as they age is also of great concern [39,54].

Our study also used self-report measures to determine the perceived QoL of the boys with DMD relative to unaffected controls. The boys with DMD reported lower physical, social and school-related scores, resulting in significantly lower total composite QoL scores. However, there was no effect of the disease in the emotional area of QoL, even as the boys aged. The differences in these responses may be due to the types of questions used in the PedsQL^TM^ assessment. The questions addressed in the physical, social, and school-related areas are based on difficulties in actual performance, such as physical activities, making friends, or missing school due to medical/physician appointments. On the other hand, the emotional subscale of the PedsQL^TM^ consists of questions about feelings of sadness, anger, and fear. These may be emotions that are evident in most children to some degree and may not necessarily be exaggerated by the disease. It is interesting to note that although QoL scores for boys with DMD were significantly lower than for unaffected boys, these scores did not notably decrease within the cohort of children with DMD when the disease has typically progressed. Other researchers have also reported no changes in QoL related to disease progression [11,12,43,55]. In a longitudinal study, Simon and colleagues (2010) used the Life Satisfaction Index for Adolescents, which they determined to be a more effective instrument for boys with DMD based on its limited focus on physical abilities. No comparisons were made with unaffected boys, but the cohort of 95 boys with DMD did not show a significant decrease and no loss of QoL was observed among the boys most affected by the progression of the disease [55]. Abresch et al. [56,57] determined that physical functioning and level of disability alone did not alter overall QoL, a finding that was also reported by Kohler et al. [43] using the Short-Form 36 of the medical outcome questionnaire. Finally, based on PedsQL^TM^ scores established and validated in a variety of patient populations [37,58], the overall scores for our cohort of boys with DMD were lower than scores from children with other major chronic health issues.

In our study, we did not find a relationship between CAPE participation scores and PedsQL^TM^ quality of life scores for unaffected boys or boys with DMD, although relationships between QoL and specific clinical measures that are typically assessed in boys with DMD have been observed. Bray et al. [13] found relationships between age, steroid use, physical functioning and QoL in DMD. McDonald et al. [44] utilized parent-proxy report and found direct relationships between the physical domains of QoL, age, and such clinical assessments as supine to stand, walk/run 10 meters and walk up 4 stairs. Findings from other studies in DMD support the thought that meaningful and developmentally important life activities, such as playing with and actively engaging with friends, may be related to QoL [11,59]. It is difficult to explain these varying results and why we did not find a correlation between participation and QoL. Although our sample size was small, it would also appear that QoL is complex and more global in nature than the straightforward and explicit report of actual participation. As Colver theorized, QoL cannot be measured directly, but only captured through a variety of questions that seek to place value on a particular variable [60]. Therefore, QoL is a concept and is subjective [55], while participation is a real life experience and is objective [61-63]. Finally, our results may differ from some studies because we chose to use child report in lieu of parent proxy report to obtain a full understanding of the children’s perception of their QoL. Literature in this area shows that parents and children often differ in their agreement of the child’s QoL [13,44,64], but the larger issue appears to be whether a child is capable of self-report [65,66]. We took the stance, as others have, that children can provide valuable information about their health and that quality of life is subjective and therefore should be a person’s individual view [33,61,65,66].

### Limitations

We acknowledge there are a few limitations to our study. Although our population of boys with DMD is rare, our sample size may be considered relatively small, especially when broken into age groups. Moreover, as this was not a longitudinal study, comparing different age groups may not be giving us information about changes as the disease progresses. Lastly, although the PedsQL^TM^ is a thoroughly developed generic measure that is brief, easy to understand, and captures information relevant to both children with a disease/illness and unaffected children, we acknowledge that the PedsQL^TM^ domains are limited, are not designed to assess a full range of functioning and are not specific to children with a neuromuscular disorder.

## Conclusions

Although a progressive disease, such as DMD, influences the physical ability to participate in many activities, it’s important to acknowledge that restrictions in participation should not inevitably arise from this or any childhood disease. Optimum management of DMD requires a multidisciplinary approach that focuses on anticipatory and preventive measures as well as active interventions to address the primary and secondary complications resulting from the disorder [6]. Such a multidisciplinary approach would benefit from using a framework to conceptualize the process of living with a progressive disease. The ICF-CY [25] provides a conceptual framework and specific terminology that facilitates investigation of the consequences of chronic illness and impairment in children and youth. The ICF model provides areas of intervention that can enhance the participation of children whose functional well-being is at risk. Interventions that focus on multiple aspects of the child and address participation afford children the opportunity to continue to engage in age and developmentally appropriate activities, and ultimately may serve to promote overall health [61,62]. Results from our study underscore the need to further evaluate activities that children elect to participate in to obtain an accurate account of a child’s level of participation and how participation changes throughout the course of a disease. Additionally, exploring how participation may relate to specific clinical tests may give us a better understanding of areas of intervention.

## Competing interests

The authors declare that they have no competing interests

## Authors’ contributions

RMB conceived and designed the study, acquired the data, analyzed and interpreted the data and drafted and revised the manuscript. CS recruited the subjects, scheduled times for the evaluations, and helped to draft and revise the manuscript. DJL participated in the design of the study and helped to draft and revise the manuscript. KV is the principal investigator of the larger study from which this pilot data was collected; KV participated in the design of the study and was involved in the drafting of the manuscript and revising for critically important intellectual content. All authors participated sufficiently in the work to take public responsibility for appropriate portions of the content. All authors read and approved the final manuscript.
